# *In vitro* analysis of CTLA-4 mediated transendocytosis by Regulatory T cells

**DOI:** 10.1007/978-1-0716-2647-4_12

**Published:** 2023-01-01

**Authors:** Erin Waters, Cayman Williams, Alan Kennedy, David M Sansom

**Affiliations:** UCL Institute of Immunity and Transplantation Pears Building, Royal Free Campus, London NW3 2PP, UK

**Keywords:** Treg, expansion, CTLA4, Transendocytosis, CD80, CD86

## Abstract

Regulatory T Cells (Treg) constitutively express the inhibitory receptor CTLA-4, which is fundamental to their role in immune suppression. Mechanistically, CTLA-4 on Tregs can attenuate T cell activation by physically removing and internalizing co-stimulatory ligands CD80 and CD86 from the surface of antigen-presenting cells by transendocytosis. Therefore, the process of transendocytosis can be harnessed as a tool to study the molecular basis of CTLA-4 biology and a key aspect of Treg suppressive function. In this chapter, we describe a method of human Treg isolation and expansion resulting in high CTLA-4 expression. We then detail a transendocytosis assay using artificial antigen presenting cells (DG-75 B Cell lines) expressing fluorescently tagged ligands mixed with the expanded Tregs. This methodology can be applied to testing of patients carrying CTLA-4 mutations, providing a robust model to assess the degree of functional disruption.

## 1 Introduction

Regulatory T cells (Tregs) are essential regulators of the immune response. Whilst Tregs possess multiple functions that mediate immune suppression, CTLA-4 function is widely appreciated as a fundamental characteristic of Treg suppressive abilities [1] Accordingly, global loss of CTLA-4 expression or conditional deletion of Treg CTLA-4 in mice results in fatal lymphoproliferative disease and systemic T cell-mediated autoimmunity [2–4]. These consequences appear to largely result from the failure of Tregs to control CD28 co-stimulation on conventional T cells (Tcon), since concurrent deletion of CD28 on CD4+ Tcon abrogates the effects of CTLA4 loss [2, 5]. Similarly, deletion or blockade of the ligands CD80 and CD86, which are shared between these receptors, also ablates this fatal lymphoproliferative phenotype [6, 7]. Thus, a significant proportion of CTLA-4 biology relates to its behaviour on Tregs.

The autoimmune protective effects of CTLA-4 are exemplified in patients where loss of function mutations in human CTLA-4 manifests as profound autoimmunity [8]. Haploinsufficiency in *CTLA4* results in a variable immunodeficiency characterised by hyperactivated effector T cells, wide ranging autoimmune features and loss of circulating B cells accompanied by hypogammaglobulinemia [9]. Genome wide association studies (GWAS) have identified *CTLA4* as an important susceptibility locus in many autoimmune diseases including Rheumatoid Arthritis, Primary Biliary Cholangitis and Type-1 Diabetes [10–12]. In addition, immune checkpoint inhibitors targeting CTLA-4 are associated with immune related adverse effects, limiting their therapeutic potential [13]. Therefore, the ability to assess CTLA-4 function in humans is of considerable importance.

Although the function of CTLA4 as a negative regulator is established, several models for its mechanism have been proposed. Originally considered to provide a cell-intrinsic inhibitory signal upon ligand binding, this does not fit well with the observed cell-extrinsic nature of CTLA4 function on Tregs in mixed bone marrow chimera models, or Tcons in TCR transgenic co-transfer models [14–16]. Mechanistically, we have demonstrated CTLA-4 can physically remove CD80 and CD86 from opposing cells and internalise them in a process termed trans-endocytosis, destroying captured ligands in lysosomes [17]. This limits the quantity of ligand available for CD28 co-stimulation, ameliorating T cell activation. This mechanism exploits the dynamic intracellular trafficking of CTLA4 as a result of clathrin-mediated endocytosis and explains its predominantly vesicular location [18]. In support, modelling of T cell:APC interactions determined the affinity bias of CTLA4>CD28 is not sufficient to control co-stimulation following low affinity self-antigen presentation to TCR on Tcons, and physical depletion of ligand was suggested to be required to prevent autoimmune responses [19]. Qualitative and quantitative characterisation of CTLA-4 transendocytosis identifies several key features of CTLA-4 biology that are integral to this mechanism. These include the quantity and timing of CTLA-4 and ligand expression, rates of CTLA-4 recycling, T cell:APC ratio and the affinities for its two ligands [20].

In this chapter, we describe protocols for the purification and *in vitro* expansion of human Tregs and their use *in vitro* assays designed to investigate the process of CTLA4-mediated transendocytosis. Application of these methods can be used to identify potential defects in CTLA-4 function as seen in patients carrying CTLA-4 mutations.

## 2 Materials

### 2.1 Purification of Regulatory T Cells (T regs)

1. Leukocyte Reduction System (LRS) cones obtained from NHS Blood and Transplant or fresh human blood sample.
2. Oxoid™ Phosphate Buffered Saline (PBS) Tablets [Thermo Scientific].
3. 50 ml conical tubes.
4. RosetteSep™ Human CD4+ T Cell Enrichment Cocktail [Stemcell Technologies].
5. Ficoll-Paque PLUS [GE Healthcare]
6. Pasteur pipettes.
7. MACs Buffer: PBS supplemented with 2 mM EDTA [Sigma-Aldrich] and 0.5% Bovine Serum Albumin (BSA).
8. CD25 MicroBeads II, human [Miltenyi Biotec].
9. LS Columns [Miltenyi Biotec].
10. MidiMACS™ Separator [Miltenyi Biotec].
11. MACS MultiStand [Miltenyi Biotec].
12. 30 ml polypropylene tubes.
13. Sort Buffer: PBS supplemented with 2mM EDTA and 25 mM HEPES.
14. Antibodies: Human anti-CD4 APC Ab, clone RPAT-4 [Biolegend], Human anti-CD25 PE Ab, clone 3-G10 [Thermofisher], Human anti-CD127, clone A019D5 [Biolegend], Human anti-CTLA4 BV786, clone BNI3 [BD Biosciences] and Human anti-FoxP3, clone 236A/E7 [Thermofisher].
15. 5 ml Round Bottom Polystyrene Test Tubes.
16. eBioscience™ Foxp3 / Transcription Factor Staining Buffer Set [Invitrogen].

### 2.2 Expansion of Regulatory T Cells (T regs)

1. DG-75 cell culture media: RPMI supplemented with 10% FBS, 2 mM L-Glutamine, 100 U/mL penicillin, and 100 mg/mL streptomycin [all from Life Technologies, Gibco].
2. T reg cell culture media (complete OpT): OpTimizer Medium, supplemented with OpTimizer T-Cell Expansion Supplement, 10% FBS, 2mM L-Glutamine, 100 U/ml penicillin, and 100 mg/ml streptomycin (all from Life Technologies, Gibco).
3. Human anti-CD3 Ab, Clone OKT3 [Functional Grade].
4. Recombinant Human IL-2 (R&D Systems).
5. Round-Bottom 96 well tissue-culture plate.

### 2.3 Transendocytosis Assay

#### Basic Protocol

1. CellTrace Violet Cell Proliferation Kit [Thermofisher Scientific].
2. T reg cell culture media: OpTimizer Medium, supplemented with OpTimizer T-Cell Expansion Supplement, 10% FBS, 2mM L-Glutamine, 100 U/ml penicillin, and 100 mg/ml streptomycin [all from Life Technologies, Gibco].
3. Human anti-CD3 Ab, Clone OKT3 [Functional Grade].
4. Recombinant Human IL-2 (R&D Systems).
5. Phosphate Buffered Saline (PBS) [Thermo Scientific].
6. 5 ml Round Bottom Polystyrene Test Tubes.

#### Time Course/Ratio

6 Round-Bottom 96 well tissue-culture plate.
7 eBioscience™ Foxp3 / Transcription Factor Staining Buffer Set [Invitrogen].

## 3 Methods

The following methods are applicable to cells derived from leukocyte enriched leukapheresis cones and to freshly isolated blood, with the numbers of Treg isolated depending on the starting material.

### 3.1 Purification of CD4+ T cells

**Figure 1:**
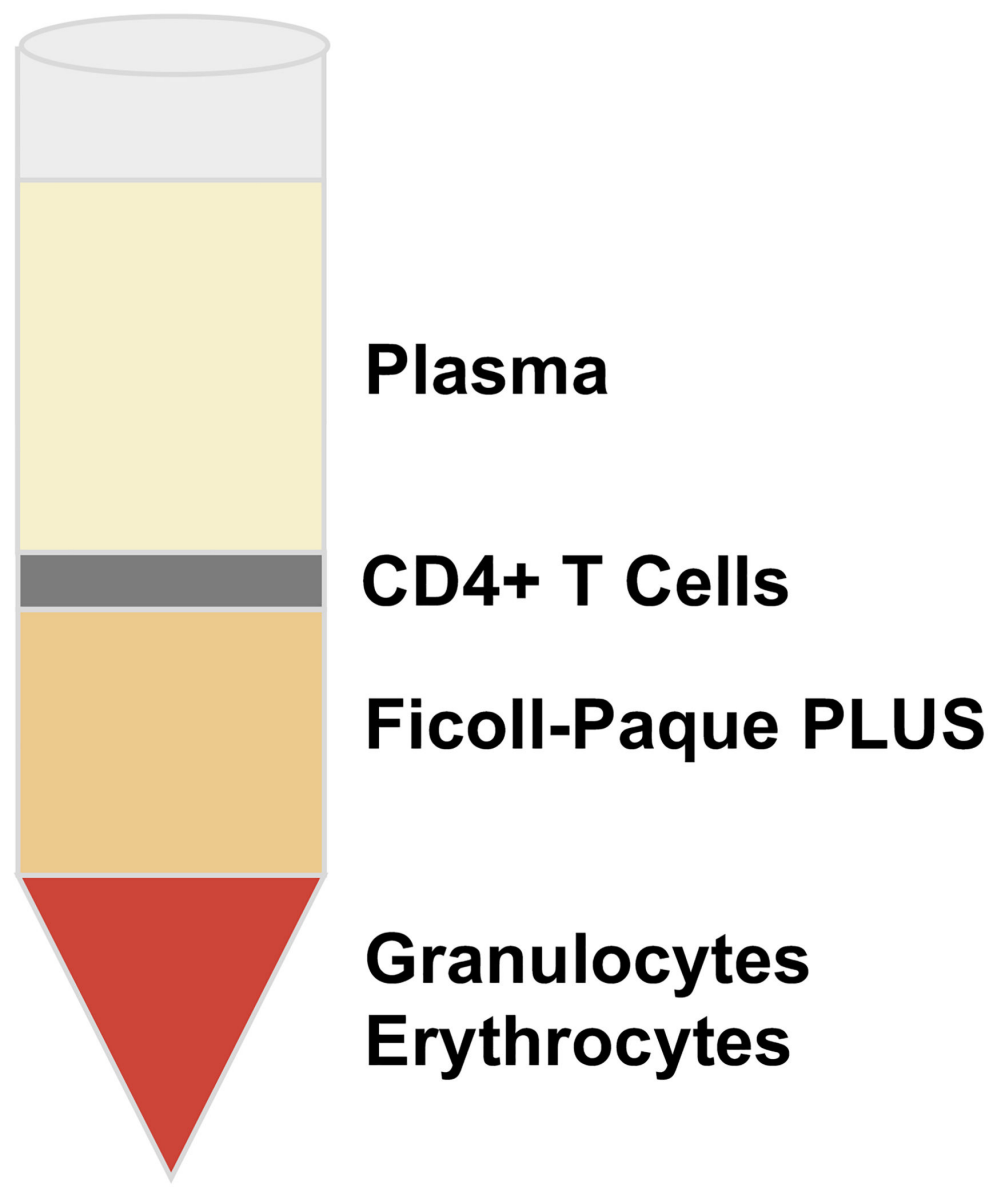
Ficoll-Paque Gradient for CD4+ T Cell enrichment. Schematic of expected layers following Ficoll Separation and treatment with RosetteSep Human CD4+ T cell enrichment cocktail of peripheral blood, or blood from a leukocyte cone.

1. Dilute sample from leukocyte cone with PBS up to a volume of 50 ml (*see* **Note 1**), or fresh peripheral blood samples 1 in 2 with PBS, in a 50 ml falcon tube.
2. Add 400 µl RosetteSep Human CD4+ T cell enrichment cocktail per 50 ml of diluted sample and gently invert to mix. Incubate for 20 min at room temperature.
3. Add 15 ml Ficoll-paque PLUS to a clean 50 ml falcon tube and layer 20-25 ml of diluted blood on top of the Ficoll (*see* **Note 2**).
4. Centrifuge layered blood for 25 min at room temperature at 1200 x g with low acceleration and brake. Following centrifugation, 4 layers will be seen; Red blood cell pellet, Ficoll-paque PLUS, CD4+ enriched T cell fraction and plasma, from bottom to top respectively (Figure 1).
5. Using a Pasteur pipette, collect the CD4+ T cell layer and transfer into a new 50 ml falcon. Dilute by addition of PBS to a total volume of 50 ml.
6. Centrifuge for 10 minutes at room temperature, 300 x g with acceleration ‘9’ and brake ‘2’. Discard the supernatant and re-suspend cells in 50 ml of pre-prepared MACS buffer.
7. Count cells (*see* **Note 3**) and re-centrifuge for 5 min at room temperature, 490 x g with acceleration ‘9’ and brake ‘9’. Discard the supernatant and re-suspend CD4+ T cells with 9 µl MACS buffer per 10 million cells. Continue to Section 3.2. immediately.

### 3.2 Purification of CD25+ Tregs from CD4+ T cells

**Figure 2:**
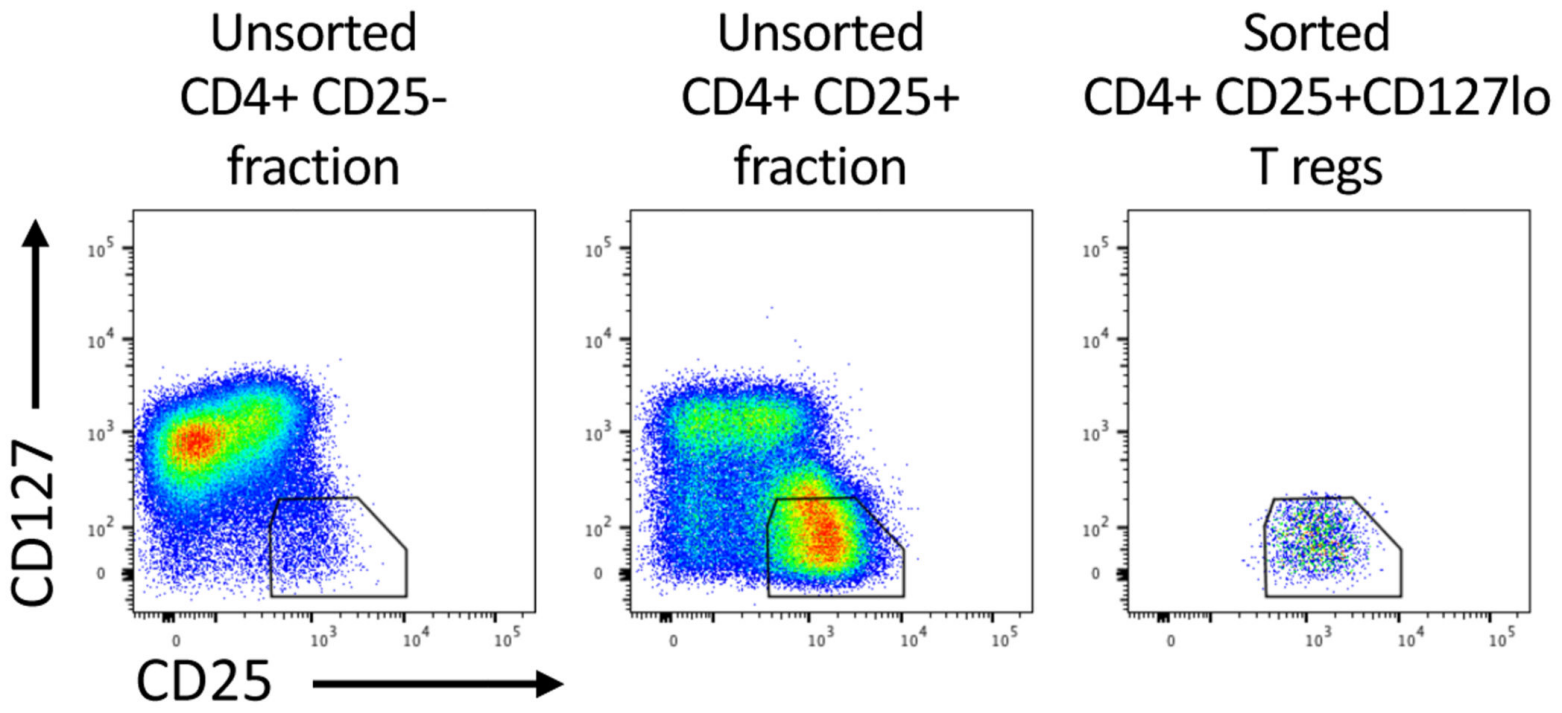
Cell sorting strategy of CD4+CD25BrightCD127lo regulatory T cells. Human CD4+ T Cells were enriched from peripheral blood. CD25- and CD25+ cell fractions were purified following CD25 Microbead II treatment and magnetic positive selection. CD25- and CD25+ fractions were stained with anti-CD4, anti-CD25 and anti-CD127 antibodies. Cells were pregated on FSC-A/SSC-A, single cells and CD4+ cells. CD4+CD25+CD127lo (Treg) cells (~54% of total) were sorted using the indicated gate.

**Figure 3:**
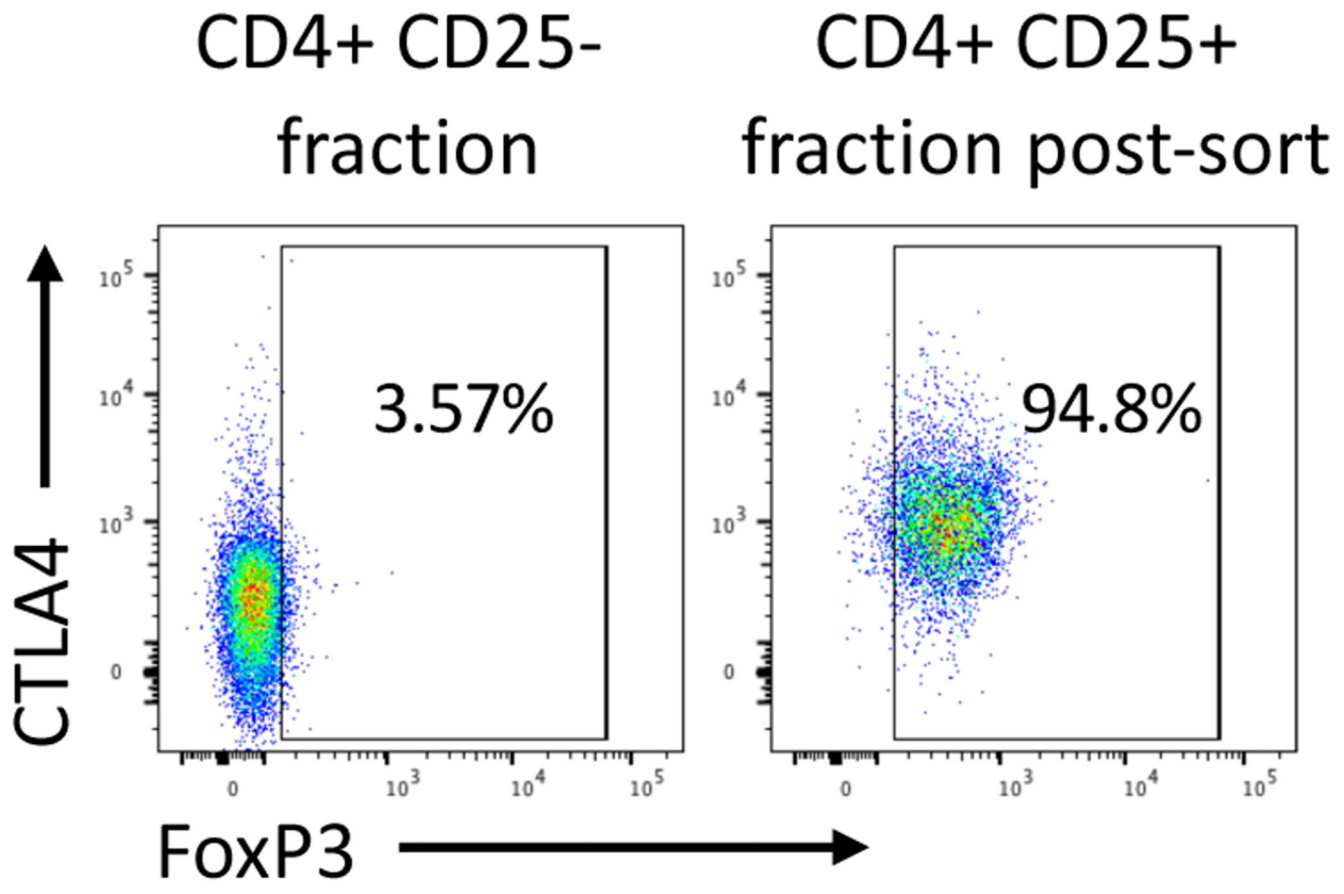
FoxP3 purity of sorted CD4+CD25+CD127lo regulatory T Cells. Human CD4+CD25+ enriched T cells were sorted based on CD25 and CD127 expression. Intracellular staining using anti-CTLA-4 and anti-FoxP3 antibodies was performed on the sorted CD4+ CD25+ fraction to determine % of FoxP3+ cells. CD4+ CD25- fraction was used as a gating control.

1. Add 10 µl CD25 Microbeads II per 100 million cells, agitate to mix and incubate at 4° for 15 min.
2. Top up with 1 ml MACS buffer per 10 million cells, or to a maximum volume of 50 ml and centrifuge for 10 minutes at room temperature, 300 x g.
3. Discard supernatant from the CD4+ T cell pellet and re-suspend in 1 ml MACS.
4. Place LS column in a magnet and wet with 3 ml MACS buffer (*see* **Note 4**). Do not let the column run dry before the addition of cells.
5. Add cell suspension to column immediately after the 3 ml wash. Unlabelled cells (CD4+ CD25-) will pass through the column (flow through), whilst magnetically labelled CD4+ CD25+ cells will remain in the column as a result of positive selection. Collect the flow through (CD4+ CD25- fraction) in a 25 ml universal.
6. After the cell suspension has run through, add an additional 3 ml MACS buffer and collect the flow through in the same tube. Repeat this wash step twice more for a total of 3 washes. This removes and collects the CD4+CD25- cells for later use.
7. After the final wash, remove the universal containing the CD4+CD25- fraction.
8. Collect CD4+CD25+ Treg cells by adding 5 ml MACS buffer to the column and removing the column from the magnet. Using the plunger, elute the positive fraction of labelled CD4+ CD25+ T cells into a fresh 25 ml universal (*see* **Note 5**).
9. Count the eluted CD4+ CD25+ cells and take an aliquot of the same number of CD4+ CD25- cells. The CD4+CD25- cells will be used to gate on the T reg population in the subsequent sorting step.
10. Wash both cell fractions by centrifuging in PBS for 5 minutes at room temperature at 490 x g. Discard the supernatant and repeat the wash step to obtain cell pellets.
11. Meanwhile, create antibody mastermix 1 (AbMM1) by addition of 4 ml CD4-APC (Clone RPAT-4, 0.2mg/ml), 6 ml CD25-PE (Clone 3-G10, 0.2mg/ml), 5 ml CD127 (Clone A019D5, 0.5mg/ml) and 150 µl of PBS.
12. Re-suspend both pellets obtained from the CD4+ CD25+ and CD4+ CD25- cell fractions in 150 µl of AbMM1 (see previous step). Incubate for 30 minutes, on ice, in the dark.
13. Add 5 ml of PBS and centrifuge for 5 minutes at room temperature at 490 x g. Discard the supernatant and repeat the wash step once.
14. Re-suspend both CD4+ CD25+ and CD4+ CD25- stained cell populations in 1 ml sort buffer (*see* materials).
15. Proceed to cell sorting. Use the CD4+CD25- cell fraction to set gates identifying the CD4+ CD25+ CD127lo Treg population. The CD4+CD25- fraction should be CD127hi. There is no need to sort on this cell fraction (Figure 2).
16. Sort CD4+ CD25+ CD127lo Treg cells from the CD4+ CD25+ enriched fraction, which will be >50% of this population (Figure 2). These are the T reg population. Retain a small aliquot of unsorted CD4+CD25-, pre-sorted CD4+CD25+ and post-sorted CD4+CD25+ fractions for completed FoxP3 and CTLA-4 phenotyping.
17. Centrifuge sorted Tregs for 5 minutes at 490 x g and resuspend at 0.4 x 10^6^ cells/ml in complete OpT media containing 2000IU of IL-2 (2X) until **step 3.3.5**.
18. Place cells retained for phenotyping (following step **3.2.15)** in 5 ml FACS tube. Add 0.5 ml PBS and centrifuge for 5 minutes at 490 x g at 4°. Discard supernatant.
19. Prepare fresh Foxp3 Fixation/Permeabilization working solution by mixing 1 part of Foxp3 Fixation/Permeabilization Concentrate with 3 parts of Foxp3 Fixation/Permeabilization Diluent (*see* **Note 6**).
20. Add 100 µl of fixation solution to each tube and incubate for 20 min on ice in the dark.
21. Centrifuge for 5 min at 490 x g at 4°. Discard supernatant and re-suspend pellet in 0.5 ml PBS. Repeat for a total of 2 washes.
22. Prepare permeabilization buffer by adding 1-part 10x permeabilization buffer in 9-parts PBS. Store on ice.
23. Re-suspend pellet in 0.5 ml of pre-chilled permeabilization buffer and centrifuge for 5 minutes at 490 x g at 4°, discard supernatant and repeat permeabilization wash for a total of 2 washes.
24. Meanwhile, create antibody mastermix 2 (AbMM2) by addition of 3 ml CTLA4 BV786 (Clone BNI3) and 3µl FoxP3 PE-Cy7 (Clone 236A/E7, 0.2mg/ml) to 50 ml permeabilization buffer per condition.
25. Add 50 ml of AbMM2 (*see* previous step) to each sample. Incubate for 1 hour, on ice, in the dark.
26. Add 0.5 ml permeabilization buffer and centrifuge for 5 minutes at 490 x g at 4°. Discard supernatant and repeat wash for a total of 2 washes.
27. Re-suspend pellet in 0.4 ml FACS buffer (*see* materials) for flow cytometry analysis. Use CD4+ CD25- fraction and unsorted CD4+ CD25+ fraction to establish gates. This is required each time as donor variation in protein levels will influence the position of the gates. Mean expected % FoxP3+ is 94.3% +/- 1.6% SD (Figure 3).

### 3.3 In vitro expansion of CD4+ CD25 bright CD127- Tregs

1. Collect a T75 flask containing CD86 transduced DG-75 human B cells grown to ~70% confluency in a 25 ml universal (*see* **Note 7**).
2. X-ray irradiate DG-75 cell line with a dose of 75Gy.
3. Centrifuge cells for 5 min at 490 x g, discard supernatant and re-suspend the pellet in 10 ml of PBS.
4. Count cells, centrifuge for 5 minutes at 490 x g, discard supernatant and re-suspend in complete OpT media at cell density required for plating.
5. Plate DG-75 and isolated T regs at a 1:1 ratio with 1 µg/ml of soluble anti-CD3 (Clone OKT3) and 1000 IU/ml of IL2 following Table 1 (*see* **Note 8**).
  For example, add 2 µg /ml (2X) of soluble anti-CD3 to a 0.4 x 10^6^ cells/ml DG-75 cell suspension, before adding 100 ul of DG-75 cells per well of a 96 U-bottom plate. Add 100 ul of T reg suspension obtained in **step 3.2.17** to the wells containing DG-75.
6. IL2 should be replenished every two days at a minimum. Gently remove 50 µl of culture media from the well and replenish with IL2 in OpT to re-establish a final concentration of 1000IU/ml IL2 (*see* **Note 9 & 10**).
7. At day 3-5 post-isolation, re-suspend and transfer half the culture into fresh wells and add complete OpT with IL2 to re-establish a working concentration of 1000 IU/ml IL2. Splitting the Tregs may occur between days 3 and 5 depending on the proliferative response of the cells which is donor dependant.
8. At day 7 post-culture, re-stimulate Tregs. To do this, pool T regs and centrifuge for 5 min at 490 x g to pellet. Discard supernatant and gently re-suspend in 5 ml of complete OpT containing 1000 IU IL2.
9. Count T reg yield. Expansion is donor dependant, however a ~2-4 fold expansion is usually observed by day 7.
10. Centrifuge for 5 minutes at 490 x g, discard supernatant and re-suspend in complete OpT containing 2000 IU IL2 (2X). At this point, take an aliquot of Tregs to check the FoxP3 purity of the cells by flow cytometry as described in section 3.2.
11. Follow **steps 3.3.1-5** providing the required TCR and CD28 (co-stimulatory) activation for re-stimulation.
12. Continue to replenish IL2 every two days and split Tregs as required, following culture plate layouts depicted in Table 1 as T regs proliferate.

### 3.4 CTLA-4 Transendocytosis assays

**Figure 4:**
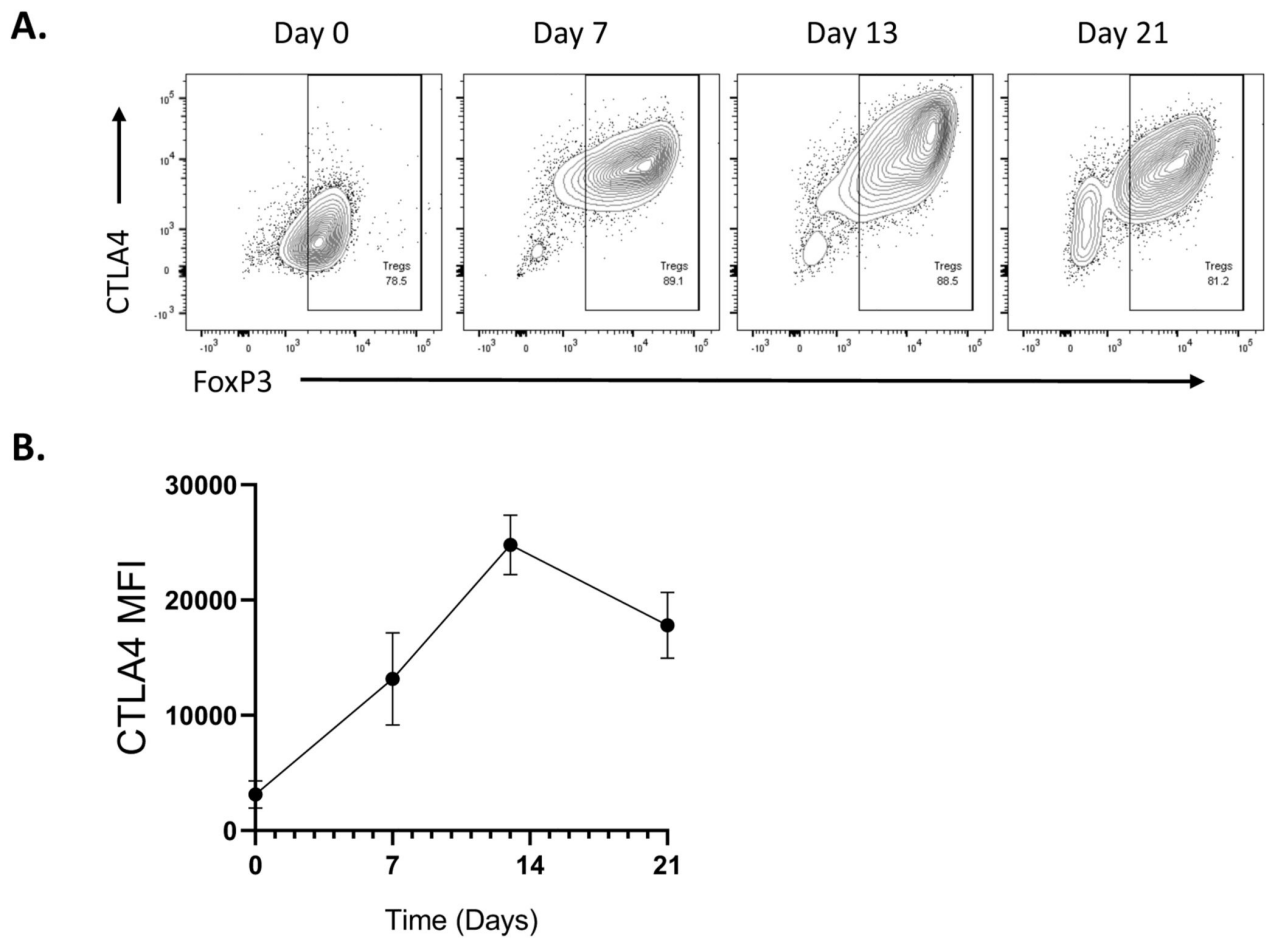
CTLA-4 expression of expanded regulatory T Cells (Tregs) over time. Human Tregs were purified from leukocyte cones. Tregs were stimulated with 100ng/ml of anti-CD3 at a 1:1 ratio of DG-75 B Cells expressing CD86 ligand in the presence of 1000 IU IL2. IL2 was replenished every 2 days and Tregs were re-stimulated every 7 days. **A**. CTLA-4 expression was determined by flow cytometry at days indicated. **B**. Graphical representation of CTLA-4 mean fluorescence intensity from 3 independent donors at indicated time points.

To study CTLA-4 mediated transendocytosis, we use expanded CTLA-4 expressing Tregs obtained in Section 3.3 at day 12 post isolation. At day 12, the CTLA-4 expression and FoxP3 purity is high and Treg numbers are plentiful (Figure 4).

**Figure 5:**
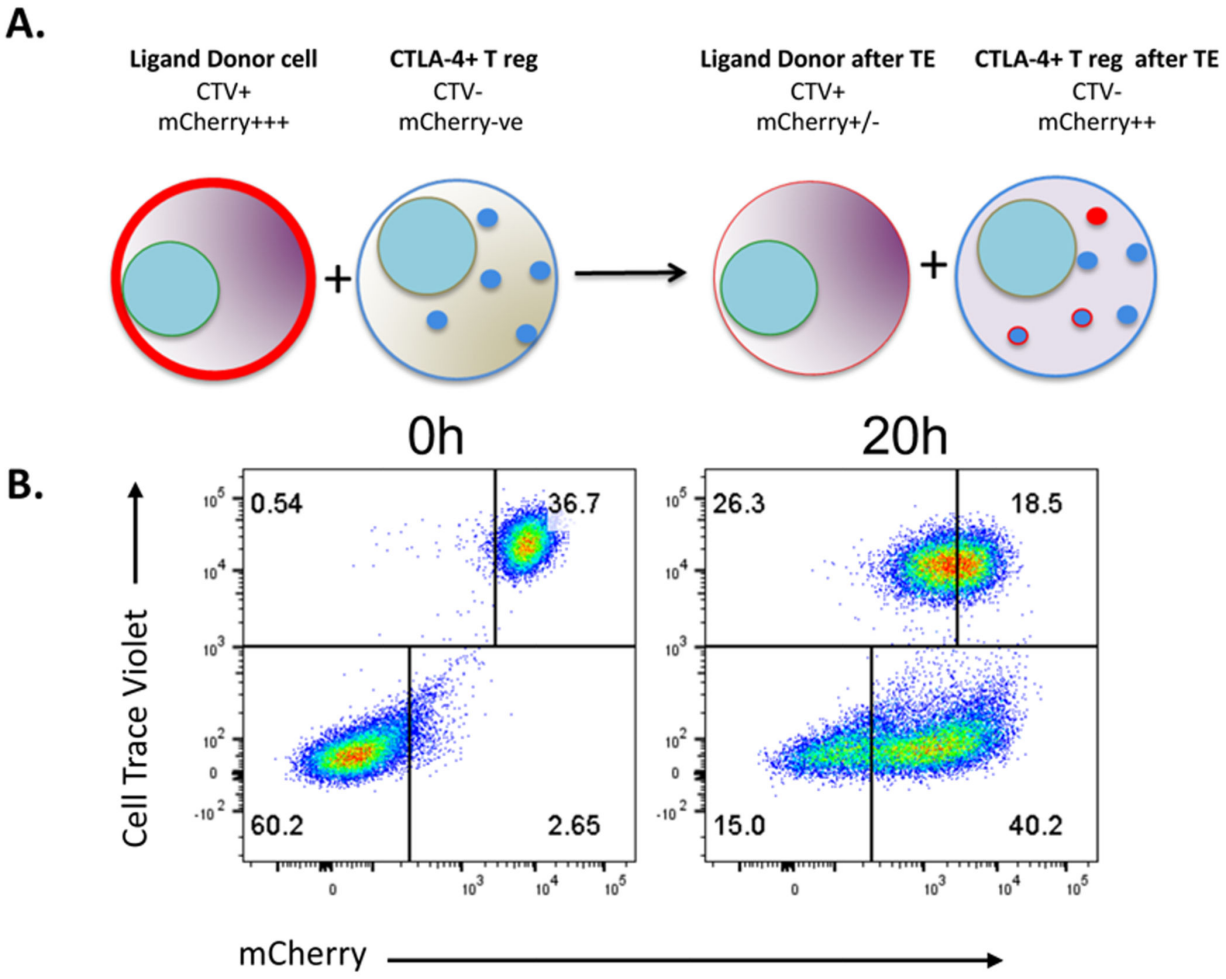
Overview of primary transendocytosis assay. **A**. Cartoon depiction of regulatory T cell (Treg) mediated transendocytosis (TE). DG-75 B Cells expressing mCherry tagged CTLA-4 ligands CD80 or CD86 are labelled with CellTrace violet (CTV). They are mixed with purified human Tregs (CTV-). As TE occurs, CTLA-4 expressed on Tregs acquires mCherry tagged surface ligand and CTV+ cells lose mCherry expression, whilst CTV- cells gain mCherry. **B**. Flow Cytometry analysis of assay set up at a 2:1 Treg:DG-75 ratio showing CTV+mCherry+ DG-75 (upper right quadrant) and CTV-mCherry-Tregs (lower left quadrant) at 0h, with mCherry acquisition into Tregs after 20 h TE.

In general principle, the experimental set-up involves co-culture of Tregs with DG-75 B cells that have been transduced with CD80 or CD86, mimicking an antigen-presenting cell (APC), prior to analysis by Flow Cytometry. Fluorescently tagged ligands (e.g. GFP or mCherry) are used to detect ligand transfer and avoid the issue of impaired ligand detection by antibody staining due to CTLA-4 binding. DG-75 B Cells are also labelled using an intracellular dye, permitting easy identification of both Tregs and APCs (DG-75) (Figure 5). This set-up enables transendocytosis to be quantified by measuring both ligand depletion from the DG-75, and ligand uptake into the Treg.

#### 3.4.1 Labelling of DG-75 ligand expressing lines with Cell-Trace Violet

1. Dilute 5 mM CellTrace Violet (CTV) stock solution to a working concentration of 5 µM by performing a 1:1000 dilution in PBS, allowing 1 ml per 1 x 10^6^ DG-75 cells.
2. Collect cells by centrifugation at 500 x g for 4 min. Wash cell suspension once with PBS to remove residual culture medium. Re-pellet cell suspension by centrifugation and remove supernatant.
3. Gently resuspend cells in 1 ml of PBS containing CTV per 1 x 10^6^ cells.
4. Incubate at 37°C for 20 min, protected from light. Give a gentle shake halfway through incubation.
5. Add 5 ml of complete culture medium to the cells and incubate for a further 10 min to quench the dye.
6. Centrifuge for 4 min at 1600 x rpm and wash once in 5 ml complete culture medium. Count cells in between wash steps, and resuspend cells at 2 x 10^6^ cells/ml.

#### 3.4.2 Co-culture of DG-75 with Tregs to observe trans-endocytosis

1. Create a 4000 IU (4X) stock solution of IL2 containing complete OpT medium and seed 50 µl per well as per desired layout into a 96-well round-bottom plate.
2. Create a 400 ng/ml (4X) stock solution of anti-CD3 (clone OKT3) containing complete OpT medium and add 50 µl to each IL2 containing well.
3. Dilute T reg suspensions in complete OpT medium to a concentration of 2 x 10^6^ cells/ml.
4. Add 50 µl of Tregs and 50 µl of CTV labelled DG-75 to each reagent containing well. This gives a 1:1 ratio of Treg to DG-75 and a total of 0.2 x 10^6^ cells in 200 µl volume, containing 100 ng/ml anti-CD3 and 1000 IU of IL-2 per condition.
5. Ensure each well is thoroughly mixed by gently resuspending using a 200 µl pipette.
6. Incubate at 37°C, 5% CO_2_ overnight (16-24) h.
7. After incubation, harvest cells into FACs tubes and place on ice for immediate acquisition (*see* **note 11**) or follow the fixation and permeabilization protocol detailed in section 3.5.

Adaptations of this general protocol can be used to refine experimental questions and are outlined in subsequent subheadings. Consequently, the assay protocol described above provides a foundation upon which multiple fundamental characteristics of trans-endocytosis can be investigated. (e.g. comparing the impact of CD80 and CD86, time, APC numbers etc.), control groups (no ligand) are standard practice and should be included in every experimental setup.

Control groups for trans-endocytosis assays:

- 0.2 x 10^6^ Tregs alone (baseline Treg with no trans-endocytosis), with and without IL2 and OKT3.
- 0.2 x 10^6^ each DG-75 cell line alone with and without IL2 and OKT3 (baseline ligand with no trans-endocytosis).
- Tregs + DG-75 which don’t express ligand with and without IL2 and OKT3 (negative control).

### 3.5 Effect of time on CTLA-4 mediated trans-endocytosis

Trans-endocytosis by CTLA-4 is highly dependent on features such as CTLA-4 recycling and Treg:APC contact times. As such the process is spatiotemporal in nature, and it is therefore of interest to study this kinetic with time as a continuous variable.

1. Set up assay as in Section 3.4. but use one 96-well plate per timepoint; 0 – 24 hours.
2. At each desired time point, centrifuge plate at 490 x g for 5 min to pellet cells. Flick plate to discard supernatant. Wash cells by addition of 200 µl PBS per well and repeat the centrifugation step.
3. Prepare fresh Foxp3 Fixation/Permeabilization working solution by mixing 1 part of Foxp3 Fixation/Permeabilization Concentrate with 3 parts of Foxp3 Fixation/Permeabilization Diluent.
4. Add 100 µl of fixation solution to each well and incubate for 20 min on ice in the dark.
5. Centrifuge at 490 x g for 5 min, then wash twice in 200 µl of PBS before resuspending in 200 µl of FACs buffer and storing at 4 °C.
6. Once all time-points are completed, samples can either be acquired and analysed for CTV and GFP/mCherry (ligand) or subjected to further phenotyping by antibody staining at the experimenters will.

### 3.6 Effect of Treg:APC ratio on CTLA-4 mediated trans-endocytosis

**Figure 6:**
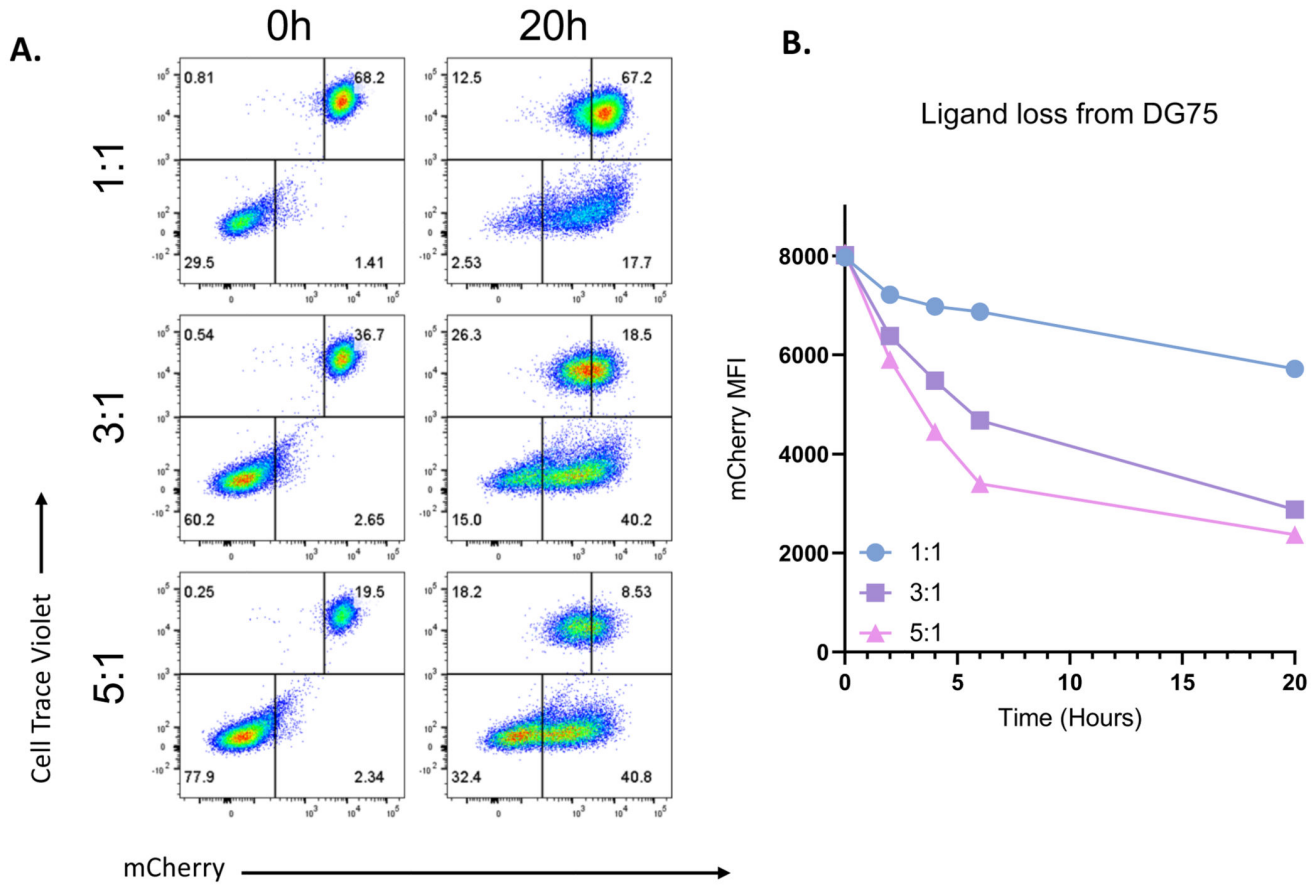
Impact of Treg:DG-75 ratio on efficiency of transendocytosis. **A**. Flow Cytometry analysis of assay set up at a 1:1, 3:1 and 5:1 Treg:DG-75 ratio, at 0h and 20h timepoints. Plots display CTV+CD80-mCherry+ DG-75 (upper right quadrant) and CTV-mCherry-Tregs (lower left quadrant) at 0h. CD80-mCherry loss from CTV+ DG-75 and acquisition into CTV- Tregs is observed after 20 h TE. B. Time course of CD80-mCherry depletion from DG-75 lines at varying ratios. Data shown is raw mean fluorescence intensity (MFI) gated on CTV+ populations.

Increasing the ratio of Tregs to APCs results in excess CTLA-4 relative to ligand, focusing the assay towards ligand loss as quantitative measure of trans-endocytosis efficiency (Figure 6). Conversely, increasing the ratio of APCs to Tregs saturates CTLA-4, enhancing ligand uptake and increasing detection sensitivity. Therefore, it is of interest to adjust the Treg:APC ratio dependant on the experimental aim.

1. Set up assay as in Section 3.4, but at step 4, varying the volume of Treg and DG-75 cell suspensions to achieve desired ratio as per table 2.

### 4 Notes

1. To maximize cell recovery from a leukocyte cone, use sterile scissors to cut bottom tubing leaving 2-3 cm attached to the cone and place above a 50 ml falcon. Cut top tubing, leaving 1 cm attached. Gently insert a 200 uL pipette tip into the top tube and release the tip. Use a 10 ml stripette to thoroughly rinse the cone with PBS. Repeat the wash once.
2. To prepare the Ficoll gradient, hold the falcon containing Ficoll at a 45° angle. Using a stripette, release diluted blood onto the side of the falcon at a slow and constant pressure.
3. Due to a high expected yield, for accurate cell counting dilution is required. If using an Automated Cell Counter, dilute 1:10 in PBS prior to counting. If using a Hemocytometer, dilute 1:100 in PBS prior to counting. Multiply the cell-count by the dilution factor.
4. We recommend use of LS Columns with the MidiMACS or QuadroMACS Separator magnets. Check the ejection blocks in the gap of the magnet are attached prior to placing the LS column in the magnetic field.
5. Due to the strong magnetic field it is best to slide rather than pull the column from the magnet. Ensure you are prepared for collection prior to this step. This is because as soon as the magnetic field is removed labelled cells will elute.
6. A transcription factor staining buffer is required for optimal detection of Foxp3. The eBioscience™ Foxp3 / Transcription Factor Staining Buffer uses a formaldehyde fixative and is stated to be compatible with other intra-nuclear targets.
7. The DG-75 is a human B Cell lymphoma suspension line, cultured in complete RPMI 1640 supplemented with 10% FBS, 2mM L-Glutamine, 100U/mL penicillin and 100mg/mL streptomycin. MP71 retroviral vectors containing mCherry tagged CD86 fusion proteins were used to make retroviral supernatants, prior to transduction into DG-75 lines. DG-75 lines expressing CD80, or mutants of both CD86 and CD80 could be substituted/added to this protocol.
8. A yield of 0.8-2 x 10^6^ Tregs is expected from one LRS cone. This number can vary based on donor differences, the freshness of the sample and the experimental technique followed to isolate. If the initial yield is > 1 x 10^6^ Tregs then scale up culture according to Table 1.
9. T regs should sink towards the bottom, center of the well. When removing 50 µl of culture media, remove from the very upper well edge to avoid cell contact.
10. Approximately 10-20 µl of liquid evaporation is expected. Ensure the full volume of 200 µl is replenished by addition of 10-20 µl of complete OpT, before addition of 50 µl of complete OpT containing 4000 IU (4X) IL2.
11. Placing cells on ice will stop the trans-endocytosis process, however T regs and DG75 cells become less viable when retained on ice for long periods of time. If acquiring live cells, ensure you run by flow cytometry as soon as possible following completion to ensure viability.

## Figures and Tables

**Table 1:** Framework for plating of Tregs and DG75 B cells applicable for different tissue culture plates.

Plate Type	DG75 Cells			Treg Cells			Final Volume / well
	Concentration (cells/ml)	Volume of anti-CD3 giving 2X concentration (μL/ml)	Volume to add to plate	Concentration (cells/ml)	Volume of IL2 giving 2X concentration (μL/ml)	Volume to add to plate	
96 well	0.4 x 10^6^	0.2	100μL	0.4 x 10^6^	2	100μL	200 μL
48 well	0.4 x 10^6^	0.2	250μL	0.4 x 10^6^	2	250μL	500 μL
24 well	0.4 x 10^6^	0.2	500μL	0.4 x 10^6^	2	500μL	1 mL
12 well	0.4 x 10^6^	0.2	1mL	0.4 x 10^6^	2	1mL	2 mL
6 well	0.4 x 10^6^	0.2	2mL	0.4 x 10^6^	2	2mL	4 mL
Ø 10cm	0.8 x 10^6^	0.2	5mL	0.8 x 10^6^	2	5mL	10 mL
T75	1 x 10^6^	0.2	7.5mL	1 x 10^6^	2	7.5mL	15 mL
T150	1 x 10^6^	0.2	15mL	1 x 10^6^	2	15mL	30 mL

**Table 2:** Volume of cell suspensions required to alter Treg:APC ratios 4 Notes

Treg:APC Ratio	40:1	20:1	10:1	5:1	1:1	1:5	1:10	1:20	1:40
Volume of Treg (μl)	97.5	95	90	80	50	20	10	5	2.5
Volume of DG- 75 cell suspension (μl)	2.5	5	10	20	50	80	90	95	97.5
Total (μl)	100	100	100	100	100	100	100	100	100
